# Baseline behaviour moderates movement skill intervention outcomes among young children with autism spectrum disorder

**DOI:** 10.1177/13623613211009347

**Published:** 2021-04-22

**Authors:** Emily Bremer, Meghann Lloyd

**Affiliations:** 1 University of Toronto, Canada; 2 Ontario Tech University, Canada

**Keywords:** interventions – psychosocial/behavioural, movement skill, pre-school children

## Abstract

This study examined whether adaptive behaviour, emotional and behavioural challenges, and social skills, respectively, moderated the effect of a movement skill intervention on movement skills among preschool aged children with autism spectrum disorder. Twenty-seven participants with autism spectrum disorder (*N* = 13 experimental) between 3 and 5 years of age participated in a 12-week fundamental movement skill intervention. Movement skills were assessed at baseline and post-test. Adaptive behaviour, emotional and behavioural challenges, and social skills were assessed at baseline. A repeated-measures analysis of variance tested the group by time effect on movement skills. Three separate moderation analyses were then run to test the moderating effect of adaptive behaviour, emotional and behavioural challenges, and social skills, respectively, on the relationship between group assignment and movement skills at the post-test. The experimental group significantly improved their movement skills following the intervention (*p* = 0.02, ηp2=.19). The moderation analyses showed that adaptive behaviour (*b*(*SE*) = 0.86 (0.41), *p* < 0.05) and emotional and behavioural challenges (*b*(*SE*) = −1.5 (0.8), *p* = 0.06) at baseline moderated the association between group and post-test movement skills, when controlling for baseline movement skills. These findings may help caregivers and clinicians individualize treatment plans in regard to movement skill interventions for preschool-aged children with autism spectrum disorder.

**Lay abstract:**

It is common for children with autism spectrum disorder to experience delays in their movement skills. These skills are important for participation in play and physical activity. Previous research has found that movement skills can be improved with movement skill interventions. This study explored the behavioural factors of young children with autism spectrum disorder that make them most likely to improve their movement skills following a 12-week intervention. The study found that children with higher levels of adaptive behaviour and lower levels of emotional and behavioural challenges at the start of the intervention were more likely to have greater improvements in their movement skills following the intervention. These findings may help clinicians and caregivers plan which types of interventions are best suited for individual children with autism spectrum disorder.

## Introduction

Delays in motor development are increasingly recognized as a core component of autism spectrum disorder (ASD; Licari et al., 2020; May et al., 2016). Motor delays appear early in development (Lloyd et al., 2013; West, 2019) and persist through childhood and into adulthood (Bhat et al., 2011; Fournier et al., 2010). They also present across multiple facets of motor competence, more broadly, including coordination, postural stability, and sensorimotor integration (Fournier et al., 2010), leading to challenges across multiple settings. One aspect of motor competence that is consistently impacted is fundamental movement skills (Pan et al., 2009; Staples & Reid, 2010). Fundamental movement skills include locomotor (e.g. running, jumping), object control (e.g. throwing, catching), and balance or stability skills and they provide the foundation for sustained participation in a wide range of physical activities (Clark & Metcalfe, 2002). In addition to a positive association with physical activity, fundamental movement skills are also related to a range of positive health outcomes (Bremer & Cairney, 2018a; Lubans et al., 2010; Robinson et al., 2015).

Previous research has demonstrated that children with ASD exhibit fundamental movement skills that are significantly delayed (Lloyd et al., 2013; Staples & Reid, 2010). This is troublesome from both a physical health and social participation standpoint and may also present greater implications for children with ASD. Movement skills have been found to be related to social communicative skills (Hirata et al., 2014; MacDonald et al., 2013b), adaptive behaviour (Bremer & Cairney, 2018b, 2020; MacDonald et al., 2013a), and language (Bedford et al., 2016; LeBarton & Landa, 2019) among children with ASD. As these are all priority areas for intervention among this population (Provenzani et al., 2020), their association with movement skills cannot be overlooked.

Children do not gain proficiency in fundamental movement skills through free play alone; these skills need to be taught through direct instruction and developmentally appropriate activities (Logan et al., 2012; Veldman et al., 2016). There is evidence to suggest that movement skills can be improved through intervention among children with ASD (Bremer et al., 2015; Bremer & Lloyd, 2016; Ketcheson et al., 2017). For example, a recent meta-analysis by Case and Yun (2019) found a large effect of intervention on gross motor outcomes. However, this evidence is still limited in a number of ways. First, only 18 studies were included in this most recent review (only 11 of which were fundamental movement skill interventions), highlighting the limited work in this area. Second, the majority of this work has been completed with school-aged children, leaving a critical gap in the early childhood period. Early childhood is a time when developing movement competence is essential, setting children on an optimal path of development and providing opportunities to engage in active play (Veldman et al., 2016). In contrast, if movement competence is not developed during this time, children risk missing out on opportunities to engage in play – a social pursuit – and risk falling further behind their peers upon school entry.

While the research is limited, it appears that movement skill interventions can have a positive effect on movement skills among children with ASD in early childhood (Bremer et al., 2015; Bremer & Lloyd, 2016; Ketcheson et al., 2017). However, little is known regarding the baseline characteristics of participants who may be most likely to benefit from this type of intervention. Understanding the characteristics of children who may benefit the most from different types of interventions may help to best direct resources (e.g. time, money, parental expectations) into interventions that are most likely to result in favourable outcomes. This may be even more critical during the early years when caregivers and clinicians of children with recent ASD diagnoses need to choose between a large range of intervention options. Evidence is critically needed to guide clinical decisions regarding which children may respond the most, to certain types of interventions; this can help to individualize treatment plans and ensure that children are enrolling in interventions most likely to have a positive effect at that developmental timepoint (Case & Yun, 2019). While it is important that we do not withhold interventions due to a possible lack of response, helping caregivers and clinicians to make informed treatment decisions may also help to temper the possible detrimental effects of investing time and energy into an intervention where their child with ASD was unlikely to respond favourably in the first place.

To date, there is very little evidence on who may respond best to movement skill interventions, yet this has been suggested as a logical next step in this area of research (Case & Yun, 2019). We are aware of one study that explored the role of social functioning as a moderator to motor outcomes following a physical activity programme for children 8 to 13 years of age with ASD (Bo et al., 2019). These authors found that school-aged children with ASD, who had greater social impairments at baseline, demonstrated larger gains in the movement domain post-intervention. This finding was likely owing to the fact that participants with a high level of social impairment may have also had the most to gain in the motor domain from an intervention. However, the authors’ classification of these groups, based on sample-specific *z*-scores, poses a challenge for both practical and clinical interpretations of this effect. Moreover, we do not know if a similar effect would be found in preschool aged children with ASD.

Given the associations previously reported in the literature between movement skills and adaptive behaviour (Bremer & Cairney, 2018b; MacDonald et al., 2013a), social skills (Hirata et al., 2014; MacDonald et al., 2013b; Ohara et al., 2019), and emotional and behavioural challenges (King-Dowling et al., 2015; Piek et al., 2008), respectively, we hypothesize that these variables will moderate the effect of a movement skill intervention on movement skills. Further, these are domains that are commonly used to describe children with ASD, make up aspects of the core diagnostic criteria (American Psychiatric Association, 2013), and are some of the best indicators of optimal outcomes in this population (Farley et al., 2009). Thus, understanding their role as intervention moderators is of practical importance in clinical decision making. Therefore, the purpose of this study is to examine whether adaptive behaviour, emotional and behavioural challenges, and social skills, respectively, moderate the effect of a movement skill intervention on movement skills among preschool aged children with ASD.

## Method

### Design and procedure

A pre-post experimental design was employed to test the moderating effect of adaptive behaviour, emotional and behavioural challenges, and social skills on movement skill outcomes following a 12-week movement skill intervention (24 h of direct instruction). As part of a larger intervention study, participants were randomly assigned to either the intervention or a waitlist control group (four sets of siblings with ASD were randomized as a unit). The waitlist control design was used to ensure all participants had the opportunity to receive the intervention. For the current study, participants in the experimental and control groups completed two assessments: one before the intervention began (baseline) and one after the end of the 12-week intervention (post-test). All assessments were conducted in a university research lab by the primary investigator with trained graduate students assisting. Participants in the experimental group received a 12-week fundamental movement skill intervention for 2 h/week and the control group continued their usual routine. Ethical approval for the study was provided by the university’s research ethics board and informed written consent was obtained from the participants’ parent at the first study appointment.

### Participants

Participants were recruited through social media, the local children’s treatment centre, a local government funded child-care facility, the regional public health unit, previous research participants, and word of mouth. Children were eligible to participate if they were between 3 and 5 years of age, with a confirmed diagnosis of ASD provided by their parent or guardian. Exclusion criteria included (a) uncontrolled seizures and (b) self-injurious behaviours that were a risk to selves or others. Twenty-seven participants were recruited and no families who volunteered for the study were excluded.

### Movement skill intervention

The movement skills targeted in the intervention included 12 fundamental movement skills such as running, hopping, throwing, catching, kicking, striking, and jumping. The curricula of each of the intervention sessions (i.e. how the skills were taught) were informed by several different curricular resources including local physical education curriculum and previous interventions (Bremer et al., 2015; Bremer & Lloyd, 2016). Fundamental motor skills are the basic foundational skills needed for more complex game skills (Clark & Metcalfe, 2002) and each week focused on a different skill (e.g. kicking). Each intervention session incorporated a brief warm-up, structured instruction and practice (e.g. obstacle course), use of the skill in a game, and free-play exploration. The organization of the sessions was consistent for every session for consistency and structure, for example, warm-up was always first, followed by direct instruction, followed by games/practice and finally free play; but the skill or the exact game would differ (Reid et al., 2003). Therefore, each particular skill was taught and practised in a structured and repetitive way to provide an optimal learning environment for the participants. For example, if the skill was ‘balance’, after the warm-up, the participants would receive verbal instruction and visual demonstration of static balance skills (e.g. standing on one foot) and be given the opportunity to practice; the lesson would then progress in difficulty (e.g. standing on one foot while putting hands on head). After static balance, dynamic balance would be introduced, for example, walking across the room with a bean bag on their head. Next, an obstacle course that included walking on flat lines on the floor, low to the ground foam beams, and through hoola-hoops would be implemented. After these structured opportunities to practice their balance skills, the last 15 min of each session was an opportunity for ‘free play’. Each of the 12 skills identified had two intervention sessions (2 × 1 h/week) devoted to them for increased practice and instruction. Fidelity to the curriculum was measured by randomly video-taping one lesson and behaviour coding using the Noldus Observer Software platform. The focus was on the instructors and their fidelity to the lesson plan and good pedagogical practices, not the participants. For example, demonstrations of the skills, verbal prompts, physical prompts, modifications and adaptations, positive reinforcement, and transitions from one skill to another. Fidelity was found to be 95% in terms of how the intervention was delivered as intended by the lesson plan.

### Measures

#### Movement skills

The Test of Gross Motor Development, 2nd Edition (TGMD-2) was administered to participating children at baseline and the post-test as a direct assessment of their movement skills (Ulrich, 2000). The TGMD-2 is a commonly used standardized assessment for children 3–10 years of age and consists of 12 items split between two subtests (locomotor and object control; Ulrich, 2000). The TGMD-2 was administered by the senior researcher and video-recorded for scoring accuracy. The video-taped assessments were scored by trained graduate student research assistants who had over 90% inter-rater reliability in scoring. Due to the fact that the children had ASD, all children were given the opportunity to explore the testing room and become familiar with the researchers. All participants were given visual demonstrations of the skills as well as a chance to practice. Visual cues (e.g. lines on the floor, picture exchange cards) were used to facilitate understanding for some children and while some children needed to be redirected back to the task at hand, the skills themselves were not modified as per the testing manual (Ulrich, 2000). The TGMD-2 Gross Motor Quotient was used as a comprehensive measure of movement skill. The Gross Motor Quotient has a possible range of <70 (very poor) to >130 (very superior) and a mean of 90–110 where higher scores indicate higher skill proficiency (Ulrich, 2000). The TGMD-2 has demonstrated excellent internal consistency and test–retest reliability and has been used in research with children with ASD (Ketcheson et al., 2017; Ulrich, 2000).

#### Adaptive behaviour

The Vineland Adaptive Behaviour Scales-2 (VABS-2) parent/caregiver rating form was completed by parents at baseline. The VABS-2 adaptive behaviour composite standard score was used as a comprehensive measure of adaptive behaviour and includes the domains of communication, daily living skills, socialization, and motor skills (Sparrow et al., 2005). The composite standard score has a possible range of 20–160, a mean of 100 and a standard deviation of 15; higher scores indicate better adaptive behaviour (Sparrow et al., 2005). The VABS-2 has demonstrated excellent internal consistency and test–retest reliability (Sparrow et al., 2005) and is a commonly used assessment of adaptive behaviour among children with ASD (Provenzani et al., 2020).

#### Emotional and behaviour challenges

The Child Behaviour Checklist 1.5–5 (CBCL) was completed by parents at baseline to assess the level of emotional (internalizing) and behavioural (externalizing) challenges experienced by the participants (Achenbach & Rescorla, 2000). The preschool CBCL asks parents to rate 100 items on a 3-point scale ranging from 0 (*not true*) to 2 (*very true or often true*) along with questions about language development. The CBCL total problems *T*-score was used as an overall measure of emotional and behavioural challenges. The total problems *T*-score has a mean of 50 and a standard deviation of 10; higher scores indicate more emotional and behavioural challenges are present. The CBCL has demonstrated excellent psychometric properties including internal consistency, inter-rater reliability, and test-reliability (Achenbach & Rescorla, 2000), along with support for the CBCL factor model specifically among preschoolers with ASD (Pandolfi et al., 2009).

#### Social skills

The Social Skills Improvement System (SSIS) parent rating form was completed by parents at baseline to assess social skills (Gresham & Elliott, 2008). The SSIS is a standardized assessment used to measure a child’s social skills, competing problem behaviours and academic competence. For the purpose of this study, only the social skills subscale was included, with the social skills standard score providing a measure of overall social functioning. The standard score has a mean of 100 and standard deviation of 15; higher standard scores indicate better social functioning (Gresham & Elliott, 2008). The SSIS has demonstrated good internal consistency and test–retest reliability (Gresham & Elliott, 2008).

### Analysis

Descriptive statistics were calculated for all variables and independent samples *t*-tests and effect sizes (Cohen’s *d*) were used to assess baseline differences between the experimental and control groups. A repeated-measures analysis of variance (ANOVA) was used to test the group (experimental vs control) by time (baseline to post-test) effect on movement skills, with effect size reported as partial eta squared (ηp2). An alpha level of <0.05 was used to interpret statistical significance of the *t*-tests and ANOVA. Three separate moderation analyses were then run to test the moderating effect of adaptive behaviour, emotional and behavioural challenges, and social skills, respectively, on the relationship between group assignment and movement skills at the post-test. In each of the three models, group (control = 0, experimental = 1) was entered as an independent predictor, movement skills at the post-test was entered as the outcome variable and movement skills at baseline was entered as a covariate. Adaptive behaviour (VABS-2 Adaptive Behaviour Composite standard score), emotional and behavioural challenges (CBCL Total Problems T-score), and social skills (SSIS Social Skills standard score) at baseline were entered as the moderator in their respective models, testing their interaction with group assignment. In line with best practice for probing interactions, interaction terms that were significant at *p* < 0.10 were probed using both simple slopes and Johnson–Neyman techniques to examine the conditional effect of the focal predicator at various values of the moderator (Hayes, 2017; A. Hayes, personal communication, July 17, 2018). All analyses were completed in SPSS version 25 (IBM Corporation, 2017) and the PROCESS macro, using Model 1, was employed for the moderation analyses (Hayes, 2017).

### Community involvement statement

Community members were not involved in the development of the research questions, selection of data collection tools, or interpretation of findings.

## Results

The sample included 27 participants (*N* = 13 experimental) between 3 and 5 years of age. The experimental group consisted of 11 males and two females, while the control group included 11 males and three females (χ^2^(1) = 0.16, *p* = 0.69). There were no differences between the groups in regard to age, movement skills, adaptive behaviour, emotional and behavioural challenges or social skills at baseline (all *p* values >0.05; see Table 1). Participants in the experimental group significantly improved their movement skills from baseline to post-test when compared to the control group and this effect was large (*F* (1, 25) = 5.97, *p* = 0.02, ηp2 = 0.19).

**Table 1.** Descriptive characteristics of the sample, by group, at baseline.

Variable	Control		Intervention		*p* value	Effect size
	Mean	*SD*	Mean	*SD*		(Cohen’s *d*)
Age (months)	45.0	9.52	44.2	6.95	0.82	0.09
Gross Motor Quotient (TGMD-2)	73.6	14.5	82.9	16.3	0.13	0.60
Adaptive Behaviour (VABS-2)	78.2	11.8	75.2	9.04	0.47	0.05
Emotional & Behavioural Challenges (CBCL)	62.9	10.0	63.6	4.35	0.82	0.09
Social Skills (SSIS)	78.7	10.9	78.1	16.0	0.90	0.28

*SD*: standard deviation; TGMD-2: Test of Gross Motor Development, 2nd Edition; VABS-2: Vineland Adaptive Behaviour Scale, 2nd Edition; CBCL: Child Behaviour Checklist; SSIS: Social Skills Improvement System.

**Figure 1.**
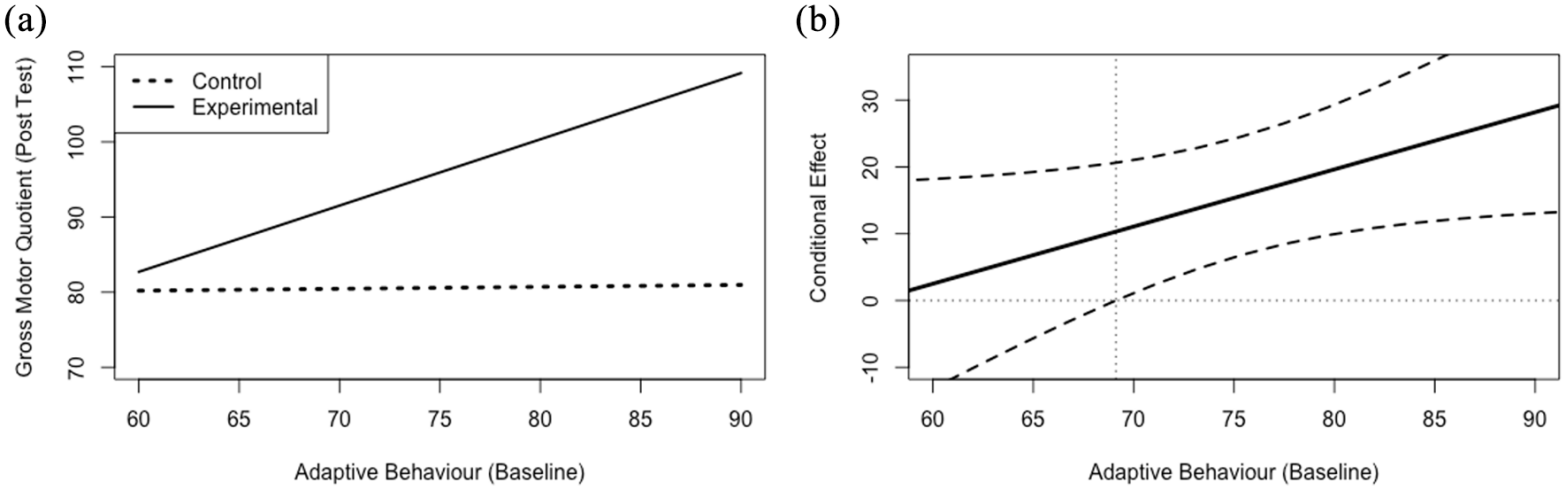
Simple slope plots (a) and regions of significance (b) for the relationship between group assignment and gross motor quotient at the post-test, while controlling for gross motor quotient at baseline, at various levels of baseline adaptive behaviour. The simple slopes (a) for the relationship between baseline adaptive behaviour and gross motor quotient at the post-test plotted by group assignment. The conditional effect (b) on the *y*-axis for the relationship between group assignment and gross motor quotient at the post-test, plotted against values of adaptive behaviour at baseline on the *x*-axis. The black dashed lines represent the upper and lower limits of the 95% confidence intervals surrounding the conditional effect (solid black line) at each level of the moderator, baseline adaptive behaviour. The vertical grey dotted line represents the value of adaptive behaviour (69.1) at which the lower limit of the confidence interval crosses the zero point and the relationship between group assignment and gross motor quotient at the post-test becomes insignificant.

Full results from the moderation analyses can be found in Table 2. Results indicated that adaptive behaviour at baseline significantly moderated (*b* (*SE*) = 0.86 (0.41), *p* < 0.05) the association between group and post-test movement skills, when controlling for baseline movement skills (Figure 1(a)). Probing of this interaction indicated that there was a conditional effect of the focal predictor (*p* < 0.05) at a VABS-2 adaptive behaviour composite score of 69.1 and higher, meaning that participation in the experimental group was only related to movement skills at the post-test among those participants scoring 69.1 or higher in adaptive behaviour at baseline (Figure 1(b)). This score roughly translates to two standard deviations below the mean in adaptive behaviour, meaning that the intervention had a positive effect on movement skills even among participants with low levels of adaptive behaviour.

**Table 2.** Moderating role of adaptive behaviour (model 1), emotional and behavioural challenges (model 2), and social skills (model 3) on the relationship between group assignment and post-test movement skills.

Predictor	Model 1 Moderator = Adaptive Behaviour Outcome = Post-test Gross Motor Quotient			Model 2 Moderator = Emotional and Behavioural Challenges Outcome = Post-test Gross Motor Quotient			Model 3 Moderator = Social Skills Outcome = Post-test Gross Motor Quotient		
	Estimate	*SE*	*p* value	Estimate	*SE*	*p* value	Estimate	*SE*	*p* value
Group	–48.8	30.8	0.13	107.4	47.7	<0.05	20.8	27.1	0.45
Baseline Gross Motor Quotient	0.52	0.16	<0.01	0.72	0.14	<0.001	0.59	0.15	<0.001
Moderator Variable	0.03	0.25	0.92	0.38	0.29	0.20	0.32	0.29	0.28
Interaction (Group × Moderator)	0.86	0.41	<0.05	–1.5	0.8	0.06	–0.07	0.34	0.83
Constant	38.3	19.2	0.06	1.5	21.1	0.94	10.1	21.9	0.65
Model Summary	*R*^2^ = 0.74, *p* < 0.001			*R*^2^ = 0.72, *p* < 0.001			*R*^2^ = 0.70, *p* < 0.001		

*SE*: standard error.

The outcome variable for each of the three models is the TGMD-2 gross motor quotient at the post-test. The moderator variable for each of the three models are as follows: Model 1: adaptive behaviour; Model 2: emotional and behavioural challenges; Model 3: social skills.

**Figure 2.**
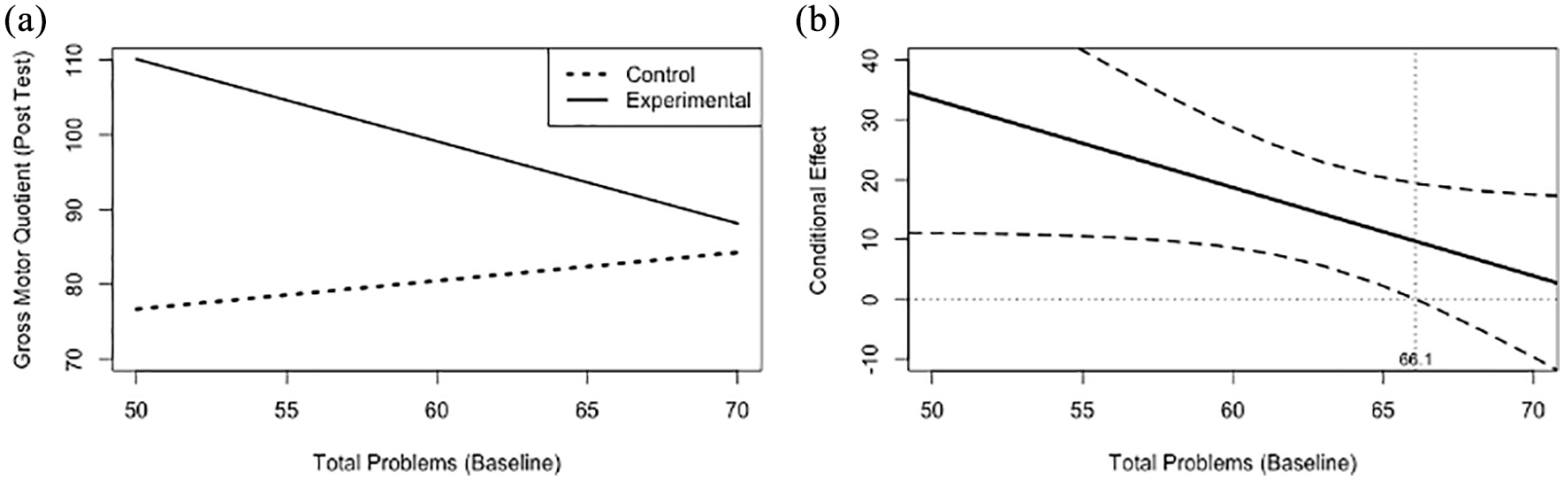
(a) Simple slope plots and (b) regions of significance for the relationship between group assignment and gross motor quotient at the post-test, while controlling for gross motor quotient at baseline, at various levels of baseline emotional and behavioural challenges (CBCL Total Problems *T*-score). The simple slopes (a) for the relationship between baseline total problems and gross motor quotient at the post test plotted by group assignment. The conditional effect (b) on the *y*-axis for the relationship between group assignment and gross motor quotient at the post-test, plotted against values of total problems at baseline on the *x*-axis. The black dashed lines represent the upper and lower limits of the 95% confidence intervals surrounding the conditional effect (solid black line) at each level of the moderator, baseline total problems. The vertical grey dotted line represents the value of total problems (66.1) at which the lower limit of the confidence interval crosses the zero point and the relationship between group assignment and gross motor quotient at the post-test becomes insignificant.

Similarly, emotional and behavioural challenges at baseline significantly moderated (*b* (*SE*) = −1.5 (0.8), *p* = 0.06) the association between group and post-test movement skills, when controlling for baseline movement skills (Figure 2(a)). Probing of this interaction identified that there was a conditional effect of the focal predictor (*p* < 0.05) at a CBCL total problems *T*-score of 66.1 and below, meaning that participation in the experimental group was only related to movement skills at the post-test among those participants scoring 66.1 or lower in emotional and behavioural challenges at baseline (Figure 2(b)). This score roughly translates to 1.5 standard deviations above the mean in emotional and behavioural challenges, meaning that the intervention had a positive effect on movement skills even among participants with elevated levels of emotional and behavioural challenges. Finally, baseline social skills (*b* (*SE*) = −0.07 (0.34), *p* > 0.10) did not moderate the association between group and post-test movement skills; therefore, this interaction was not probed further.

## Discussion

Intervention resources are often limited (e.g. financial, time, effort) for families with a child with ASD; therefore, it is imperative that decisions about which interventions are deemed to be a priority, or most likely to have an effect, are informed by evidence. Findings from this study indicate that adaptive behaviour and emotional and behavioural challenges, but not social skills, significantly moderate movement skill outcomes following a movement skill intervention. Specifically, we see that intervention effects are greatest for those participants with higher levels of adaptive behaviour and fewer emotional and behavioural challenges at baseline. Interestingly, the region of significance of these moderating effects were quite large, indicating that even participants with relatively low levels of adaptive behaviour or relatively high levels of emotional and behavioural challenges can benefit from a movement skill intervention. This finding aligns with previous research that has demonstrated large effects, overall, on movement skills following movement skill interventions for preschool aged children with ASD (Case & Yun, 2019), suggesting that the majority of children with ASD can benefit from such interventions. However, understanding the variables that moderate these effects, and to what extent, helps us to better understand which children may benefit the most from movement skill interventions.

It is not surprising that adaptive behaviour significantly moderated the intervention effect on movement skills: it is likely that a certain level of adaptive behaviour is necessary to engage in group-based, interactive interventions. Moreover, previous research has demonstrated an association between movement skills and adaptive behaviour in both early (MacDonald et al., 2013a) and middle childhood (Bremer & Cairney, 2018b). The role of adaptive behaviour as a moderator may be of particular importance. Adaptive behaviour is related to optimal outcomes among individuals with ASD (Farley et al., 2009) and is one of the most common outcome measures in ASD research (Provenzani et al., 2020), underscoring the importance of adaptive behaviour for children with ASD. Moreover, having an optimal level of adaptive behaviour may help children with ASD follow instructions and manage their behaviour within an intervention setting. Future research should, therefore, continue to explore the role of adaptive behaviour as a moderator to movement skill interventions, as well as whether adaptive behaviour can be improved through movement skill interventions.

Similarly, it makes sense that children with fewer emotional and behavioural challenges at baseline had greater improvements in movement skills following the intervention. A high level of behavioural challenges may be detrimental to a child’s participation in group-based activities, limiting their ability to engage in the activities and learn new skills. It is interesting, though, that social skills did not moderate the intervention effect on movement skills. Previous work has demonstrated a positive relationship between social skills and movement skills (Ohara et al., 2019) and that aspects of social skills can be improved following movement skill interventions (Bremer & Lloyd, 2016; Guest et al., 2017). That a certain level of social skills is not needed for gains in movement skills may suggest that in early childhood (3–5 years), one’s level of social skills at baseline does not influence intervention outcomes. However, this finding may also be due to the one-on-one instructor to child ratio provided in this intervention; meaning that a child’s level of social skill at baseline did not impact the attention or support they received throughout the study. It is possible that social skills may be more important in a setting with a higher child to instructor ratio where a child’s level of social functioning may have a greater impact on their ability to interact within the group. Regardless, we interpret this null finding as a positive: social skills represent one of the core challenges for children with ASD, especially in the early years, and these results suggest that gains in movement skills can be made regardless of one’s level of baseline social skills; and gains in motor skills can promote engagement in play and other developmentally important activities (Pellegrini & Smith, 1998; Rosenbaum, 2005). This finding is in contrast to the finding of Bo and colleagues (2019) who found that school-aged children with greater social impairments at baseline had greater improvements in movement skills following a physical activity intervention. It is possible that a moderating effect of social skills does not emerge until later childhood, or that the social skills construct measured with the SSIS does not adequately capture the social skills necessary for participation in a movement skill intervention. Therefore, the role of social skills as a potential moderator of intervention effects should be further explored.

Findings from this study may help practitioners, researchers, and caregivers to understand how to individualize treatment plans for preschool-aged children with ASD. This is a critical step in order to maximize intervention benefits and ensure that resources, such as time and money, are directed towards interventions that will have the greatest individual benefit for a child with ASD. Adaptive behaviour is relatively stable during the preschool years (Flanagan et al., 2015), and thus it may be a valuable marker by which to help make these decisions. Importantly, we found that gains in movement skills can be made for children with quite a wide range of behavioural skills, including those scoring up to 2 standard deviations below the mean in adaptive behaviour or 1.5 standard deviations above the mean in emotional and behavioural challenges, respectively. This suggests that most children with ASD can benefit from a movement skill intervention. Movement skill interventions have demonstrated a number of additional benefits for preschool-aged children with typical development, beyond gains in movement skills, including increased physical activity (Engel et al., 2018) and improved social-emotional functioning (Piek et al., 2015). Thus, motor skill interventions should routinely be included as part of the suite of interventions that young children with ASD receive (Lloyd et al., 2013). Moreover, it is critical that future research explore the impact of these interventions on outcomes other than movement skills, such as adaptive behaviour and social skills, among children with ASD.

While this study is the first to explore potential moderating effects on movement skill interventions among preschool-aged children with ASD, the findings are not without limitations. First, the sample was predominately male, limiting our ability to test potential sex effects. Although ASD is diagnosed more frequently in males than females (National Autism Spectrum Disorder Surveillance System, 2018), it is important that future work purposely explore these moderating effects in females as well. Second, the sample was relatively small and from one geographic region, which may limit our ability to generalize the findings to other populations of children with ASD. As such, future work should continue to test these moderating effects among larger, more diverse samples of children with ASD in order to replicate these preliminary findings. Third, we were limited in the diagnostic information available for our sample and did not have measures of IQ or clinical confirmation of ASD diagnosis or severity level, which may have provided further insight regarding the participants’ baseline characteristics. Finally, it is important that future research assess the long-term trajectories of these moderating effects on movement skills among children with ASD as they age.

In conclusion, findings from this study indicate that both adaptive behaviour and emotional and behavioural challenges, at baseline, moderate the effect of a movement skill intervention on the movement skills of preschool-aged children with ASD. The region of significance of these moderating effects was quite large suggesting that the majority of children with ASD can improve their movement skills following intervention. Future research should continue to explore the role of additional moderators in relation to intervention outcomes, and the long-term implications of these effects, following movement skill interventions for young children with ASD.
